# Identification of Tick-Borne Pathogens in Ticks Feeding on Humans in Turkey

**DOI:** 10.1371/journal.pntd.0003067

**Published:** 2014-08-07

**Authors:** Ömer Orkun, Zafer Karaer, Ayşe Çakmak, Serpil Nalbantoğlu

**Affiliations:** Department of Parasitology, Faculty of Veterinary Medicine, Ankara University, Ankara, Turkey; University of Texas Medical Branch, United States of America

## Abstract

**Background:**

The importance of tick-borne diseases is increasing all over the world, including Turkey. The tick-borne disease outbreaks reported in recent years and the abundance of tick species and the existence of suitable habitats increase the importance of studies related to the epidemiology of ticks and tick-borne pathogens in Turkey. The aim of this study was to investigate the presence of and to determine the infection rates of some tick-borne pathogens, including *Babesia* spp., *Borrelia burgdorferi* sensu lato and spotted fever group rickettsiae in the ticks removed from humans in different parts of Ankara.

**Methodology/Principal Findings:**

A total of 169 ticks belonging to the genus *Haemaphysalis*, *Hyalomma*, *Ixodes* and *Rhipicephalus* were collected by removing from humans in different parts of Ankara. Ticks were molecularly screened for *Babesia* spp., *Borrelia burgdorferi* sensu lato and spotted fever group rickettsiae by PCR and sequencing analysis. We detected 4 *Babesia* spp.; *B. crassa*, *B. major*, *B. occultans* and *B. rossi*, one *Borrelia* spp.; *B. burgdorferi* sensu stricto and 3 spotted fever group rickettsiae; *R. aeschlimannii*, *R. slovaca* and *R. hoogstraalii* in the tick specimens analyzed. This is the report showing the presence of *B. rossi* in a region that is out of Africa and in the host species *Ha. parva*. In addition, *B. crassa*, for which limited information is available on its distribution and vector species, and *B. occultans*, for which no conclusive information is available on its presence in Turkey, were identified in *Ha. parva* and *H. marginatum*, respectively. Two human pathogenic rickettsia species (*R. aeschlimannii* and *R. slovaca*) were detected with a high prevalence in ticks. Additionally, *B. burgdorferi* sensu stricto was detected in unusual tick species (*H. marginatum*, *H. excavatum*, *Hyalomma* spp. (nymph) and *Ha. parva*).

**Conclusions/Significance:**

This study investigates both the distribution of several tick-borne pathogens affecting humans and animals, and the presence of new tick-borne pathogens in Turkey. More epidemiological studies are warranted for *B. rossi*, which is very pathogenic for dogs, because the presented results suggest that *B. rossi* might have a wide distribution in Turkey. Furthermore, we recommend that tick-borne pathogens, especially *R. aeschlimannii*, *R. slovaca*, and *B. burgdorferi* sensu stricto, should be taken into consideration in patients who had a tick bite in Turkey.

## Author Summary

Ticks are widespread in over all Turkey. Primary tick-borne diseases (TBDs), such as theileriosis, babesiosis, and anaplasmosis, affecting animals have been known for a long time in Turkey. However, TBDs have become a major concern in humans in recent years due to the recent Crimean-Congo hemorrhagic fever outbreak in Turkey. We know that some TBDs like CCHF, Lyme borreliosis, spotted fever group rickettsiosis, babesiosis and anaplasmosis exist in this geography. However, the other diseases except for CCHF are neglected in patients. In this study, we aimed to investigate *Babesia* spp., *Borrelia burgdorferi* sensu lato and spotted fever group rickettsiae in ticks removed from humans in different parts of Ankara by using PCR and sequencing. The result of this study showed that 4 *Babesia* species, one *B. burgdorferi* sensu lato, and 3 spotted fever group rickettsia are detected in ticks. The most striking result of this study is that *B. rossi*, which is very pathogenic for dogs, was reported for the first time from a region that is out of Africa and in *Ha. parva*. Therefore, *B. rossi* should be considered in dogs in Turkey. Furthermore, we propose that *R. aeschlimannii*, *R. slovaca*, and *B. burgdorferi* sensu stricto should be taken into consideration in patients who had a tick bite in Ankara.

## Introduction

Ticks are very important vectors of diseases affecting both humans and animals. They transmit a broader range of viral, bacterial (including rickettsial) and protozoan pathogen microorganisms than any other arthropods worldwide, and are also the main reservoirs of these pathogens [1], [2]. Tick-borne diseases (TBDs) constitute a major public health concern and they are responsible for great economic losses in terms of mortality and morbidity of livestock animals worldwide [2], [3].

The importance of TBDs is increasing all over the world. We still encounter new tick-borne epidemics [4]. The best example of a recent outbreak is that the Crimean-Congo hemorrhagic fever (CCHF) outbreak that started in northern Turkey in 2002, human cases still continue to increase and the disease spreads in many regions of Turkey [5], [6]. Moreover, the incidence of significant TBDs has highly increased during the last 30 years worldwide [4]. In recent years, both new tick-borne pathogens have been described and detailed epidemiological studies have been carried out owing to the commonly used molecular techniques. Therefore, molecular tools can provide a better understanding of the epidemiology of TBDs [3]. Nevertheless, we need new studies especially on the epidemiology and diagnosis of tick-borne pathogens and the ecology of these newly recognized disease agents [7].

In Turkey, 5 TBDs have remarkable importance for humans: Crimean-Congo hemorrhagic fever, Lyme borreliosis, spotted fever group rickettsiosis, babesiosis, and anaplasmosis. However, the other diseases except for Crimean-Congo hemorrhagic fever are neglected in patients, although whole of the pathogens have been commonly detected in ticks [5], [8]–[12]. In previous studies, e.g., human pathogenic spotted fever group rickettsiae; *Rickettsia africae*, *Rickettsia aeschlimannii*, and *Rickettsia slovaca* have been remarkable detected in ticks collected from different regions [10], [12]; however, no clinical cases have been reported in Turkey so far. Nonetheless, the studies demonstrated that the tick species which belonged to the genera *Dermacentor*, *Haemaphysalis*, *Hyalomma*, *Ixodes* and *Rhipicephalus* are responsible for major human tick bite cases and these species have a wide distribution in Turkey [12]–[17].

In this study, we aimed to investigate the presence *Babesia* spp., *Borrelia burgdorferi* sensu lato and spotted fever group rickettsiae in ticks obtained from hospitals of Ankara.

## Materials and Methods

### Collection of tick samples and morphological identification

**Figure 1:**
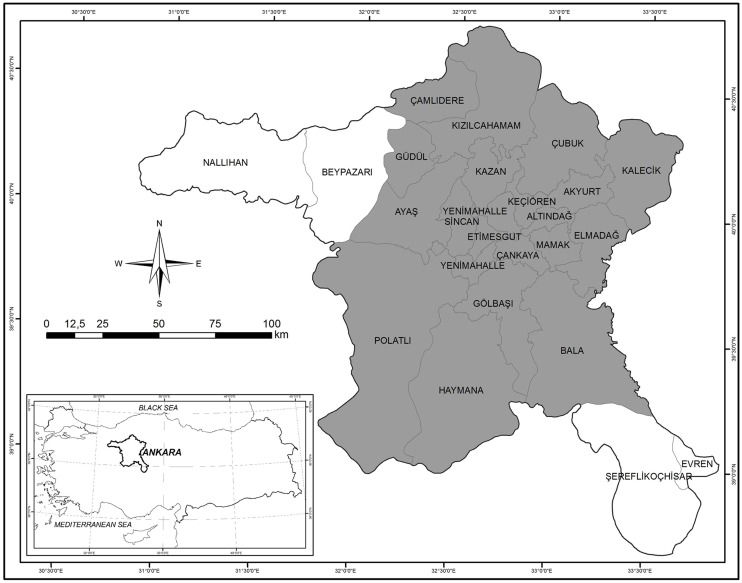
Map showing the location of Ankara province and study area.

This study was conducted in Ankara, the capital city of Turkey. Ankara province is located in Central Anatolia (Fig. 1) and is the second largest city in the county. Ankara is about 24.500 km^2^ and the population of the city is close to 5 million. Half of the total area (approximately 12.000 km^2^) is used as agricultural land. Different habitats; forest, steppe, wetlands, and salty soils are encountered in Ankara. The average annual precipitation is 242–612 mm., while the average annual temperature is 10.3–14.7°C. Ankara is under the influence of semi-arid and very cold Mediterranean climate. A large part of the province is covered with steppe. The elevation is between 550 and 2000 meters [18]. In rural areas, cattle and sheep breeding are commonly made partially intensive but mostly in pasturelands. Goat breeding is limited to some villages and goats are generally pastured while mixed with sheep. Wild animals such as wild boar, hare, fox, and ground-feeding birds (partridge, crow etc.), which are also called amplifying hosts for ticks, are abundant throughout the province.

From March 2012 to March 2013, tick specimens were obtained from humans who presented to hospitals with tick bites. Tick samples that are brought for species identification were delivered from patients with tick bites and information about the tick bite area was individually recorded carefully. Ticks were identified according to the taxonomic keys of Walker et al., 2000; Apanaskevich, 2003; Estrada-Pena et al., 2004 [19]–[21]. Engorged nymphs were incubated under the suitable conditions to allow molt to the adult stage.

### DNA extraction and PCR amplification

Each tick was first washed in 70% alcohol, then rinsed in sterile water and dried on sterile filter paper in order to avoid contamination. Ticks were individually homogenized by crushing with liquid nitrogen in a mini-mortar. DNA was individually extracted from crushed ticks by using the Qiagen DNeasy blood and tissue kit (Qiagen, Hilden, Germany) according to the manufacturer's instructions. First, an initial PCR targeted tick *16S rDNA* as internal control gene with 16S+1 and 16S-1 primers, which amplify approximately 460 base pairs (bp) fragment was performed to every tick in order to determine whether PCR inhibition [22]. Only the positive samples were further analyzed for the presence of *Babesia* spp., *Borrelia* spp., and *Rickettsia* spp.

A *Babesia* spp. genus-specific PCR was performed to each tick by using primers BJ1 and BN2, which amplify a 411–452-bp fragment of the 18S ribosomal RNA (*18S rRNA*) gene [23]. For the detection of *B. burgdorferi* sensu lato species, a nested PCR, which amplifies approximately 200-bp fragment of the *5S-23S rDNA* intergenic spacer (IGS) was carried out by using two set primers RIS1 and RIS2, and RIS3 and RIS4 for the first-step and the nested-PCR, respectively [8], [24]. Rickettsial DNA was detected by PCR using the primers *Rp* CS.409d and *Rp* CS.1258n, which amplify a 750-bp fragment of the citrate synthase gene (*gltA*) of *Rickettsia* spp. [25]. Additionally, each tick positive for *gltA* was also tested for the *ompA* gene of *Rickettsia* spp. using the primers Rr. 190.70 and Rr. 190.701, which amplify a 629–632-bp fragment [26]. DNase-RNase-free water was used as a negative control and positive controls (DNA from *B. bigemina*, *B. divergens*, *R. montanensis*, *B. burgdorferi* sensu lato) were included in all reactions. We have also performed pre-PCR with positive controls in different dilutions (1-1/100) to avoid false negative results that may occur due to low copies of bacterial and protozoan genes.

### Sequencing and phylogenetic analysis

Successfully amplified product was purified using the QIAquick Extraction Kit (Qiagen GmbH). Purified DNA was sequenced using BigDye Terminator V3.1 Cycle Sequencing Kit (Applied Biosystems, Foster City, USA). Automated fluorescence sequencing was performed with an ABI PRISM 3100 Genetic Analyzer (Applied Biosystems). Nucleotide sequences were processed using nucleotide BLAST (National Center for Biotechnology Information, www.ncbi.nlmn.nih.gov/BLAST). Sequences were edited and aligned by using BioEdit software [27]. We used jModeltest version 0.1.1 [28] to determine the most appropriate model for our data. Phylogenetic and molecular evolutionary analyses were performed by using MEGA version 5.2.1 [29]. The gene sequences obtained in this study have been deposited in GenBank under the accession numbers KF791205 to KF791260.

### Accession numbers

KF791205 *Babesia crassa* isolate BB-59/Ank-Ha. parva

KF791206 *Babesia major* isolate BB-86/Ank-Ha.punct

KF791207 *Babesia occultans* isolate BB-97/Kalecik-H. marg

KF791208 *Babesia rossi* isolate Ext-165/Ank-Ha. parva

KF791209 *Rickettsia hoogstraalii* isolate BB-56/Ank-Ha.parva

KF791210 *Rickettsia hoogstraalii* isolate BB-57/Ank-Ha.parva

KF791211 *Rickettsia hoogstraalii* isolate BB-58/Cubuk-Ha.parva

KF791212 *Rickettsia hoogstraalii* isolate BB-59/Ank-Ha.parva

KF791213 *Rickettsia hoogstraalii* isolate BB-75/Ank-Ha.parva

KF791214 *Rickettsia hoogstraalii* isolate BB-76/Ank-Ha.parva

KF791215 *Rickettsia hoogstraalii* isolate BB-77/Ank-Ha.parva

KF791216 *Rickettsia hoogstraalii* isolate BB-78/Ank-Ha.parva

KF791217 *Rickettsia hoogstraalii* isolate BB-80/Ank-Ha.parva

KF791218 *Rickettsia hoogstraalii* isolate BB-81/Ank-Ha.parva

KF791219 *Rickettsia hoogstraalii* isolate BB-82/Ank-Ha.parva

KF791220 *Rickettsia hoogstraalii* isolate BB-83/Ank-Ha.parva

KF791221 *Rickettsia hoogstraalii* isolate BB-84/Ayas-Ha.parva

KF791222 *Rickettsia hoogstraalii* isolate BB-85/Ank-Ha.parva

KF791223 *Rickettsia hoogstraalii* isolate BB-132/Ank-Ha.parva

KF791224 *Rickettsia hoogstraalii* isolate BB-133/Ank-Ha.parva

KF791225 *Rickettsia hoogstraalii* isolate BB-134/Golbasi-Ha.parva

KF791226 *Rickettsia hoogstraalii* isolate BB-135/Ank-Ha.parva

KF791227 *Rickettsia hoogstraalii* isolate BB-136/Ank-Ha.parva

KF791228 *Rickettsia hoogstraalii* isolate BB-137/Ank-Ha.parva

KF791229 *Rickettsia hoogstraalii* isolate BB-138/Akyurt-Ha.parva

KF791230 *Rickettsia hoogstraalii* isolate BB-139/Ank-Ha.parva

KF791231 *Rickettsia slovaca* isolate BB-1/Ank-D.marg

KF791232 *Rickettsia slovaca* isolate BB-2/Ank-D.marg

KF791233 *Rickettsia slovaca* isolate BB-3/Ank-D.marg

KF791234 *Rickettsia slovaca* isolate BB-50/Elma-D.marg

KF791235 *Rickettsia slovaca* isolate BB-51/Akyurt-D.marg

KF791236 *Rickettsia slovaca* isolate BB-52/Ank-D.marg

KF791237 *Rickettsia slovaca* isolate BB-66/Kızıl-D.marg

KF791238 *Rickettsia slovaca* isolate BB-67/Ank-D.marg

KF791239 *Rickettsia slovaca* isolate BB-68/Cubuk-D.marg

KF791240 *Rickettsia slovaca* isolate BB-70/Ank-D.marg

KF791241 *Rickettsia slovaca* isolate BB-72/Ank-D.marg

KF791242 *Rickettsia slovaca* isolate BB-73/Ank-D.marg

KF791243 *Rickettsia slovaca* isolate BB-74/Golbasi-D.marg

KF791244 *Rickettsia slovaca* isolate BB-129/Ank-D.marg

KF791245 *Rickettsia slovaca* isolate BB-130/Ank-D.marg

KF791246 *Rickettsia slovaca* isolate BB-131/Polat-D.marg

KF791247 *Rickettsia aeschlimannii* isolate BB-13/Bala-H.marg

KF791248 *Rickettsia aeschlimannii* isolate BB-14/Kızıl-H.marg

KF791249 *Rickettsia aeschlimannii* isolate BB-16/Ank-H.marg

KF791250 *Rickettsia aeschlimannii* isolate BB-17/Ank-H.marg

KF791251 *Rickettsia aeschlimannii* isolate BB-35/Caml-H.marg

KF791252 *Rickettsia aeschlimannii* isolate BB-46/Ank-H.aegp

KF791253 *Rickettsia aeschlimannii* isolate BB-90/Ank-H.exca

KF791254 *Rickettsia aeschlimannii* isolate BB-93/Ank-H.aegp

KF791255 *Borrelia burgdorferi sensu stricto* isolate Ext-42/Cubuk- H. marg

KF791256 *Borrelia burgdorferi sensu stricto* isolate Ext-87/Ank-H.nymph

KF791257 *Borrelia burgdorferi sensu stricto* isolate Ext-88/Ank-H.nymph

KF791258 *Borrelia burgdorferi sensu stricto* isolate Ext-106/H. exca

KF791259 *Borrelia burgdorferi sensu stricto* isolate Ext-164/Ank-Ha.parva

KF791260 *Borrelia burgdorferi sensu stricto* isolate Ext-166/Ank-Ha.parva

## Results

Localities of the tick bite were registered in Akyurt, Ayaş, Bala, Çamlıdere, Çubuk, Elmadağ, Gölbaşı, Güdül, Haymana, Kalecik, Kazan, Kızılcahamam, Polatlı, and central districts of Ankara (Table 1). The districts where ticks were collected are marked with grey on the map (Fig. 1).

**Table 1:** Tested tick species collected from different localities of Ankara: Genders and PCR positivity.

Tick species (no. tested specimens)	Localities, genders and numbers of the ticks collected	No. PCR-positive ticks		
		*Babesia* spp.	*Borrelia* spp.	*Rickettsia* spp.
***Hyalomma marginatum*** ** (30)**	Central (7M*, 3F)	-	-	1M, 1F
	Çubuk (5M, 1F)	-	1M	-
	Kazan (2M, 2F)	-	-	-
	Kalecik (1M, 2F)	1M	-	-
	Kızılcahamam (3M)	-	-	1M
	Akyurt (2M)	-	-	-
	Çamlıdere (1F)	-	-	1F
	Bala (1F)	-	-	1F
***Hyalomma excavatum*** ** (17)**	Central (5M, 4F)	-	1M	1M
	Ayaş (1M, 1F)	-	-	-
	Kalecik (1M, 1F)	-	-	-
	Akyurt (1M)	-	-	-
	Çubuk (1F*)	-	-	-
	Kazan (1F)	-	-	-
	Kızılcahamam (1M)	-	-	-
***Hyalomma aegyptium*** ** (16)**	Central (5M*, 7F*)	-	-	1F*
	Kazan (2M)	-	-	1M
	Akyurt (1F*)	-	-	-
	Kızılcahamam (1M*)	-	-	-
***Hyalomma*** ** spp. (nymph) (6)**	Central (4N)	-	2N	-
	Ayaş (2N)	-	-	-
***Dermacentor marginatus*** ** (25)**	Central (9M, 8F)	-	-	4M, 6F
	Çubuk (3M)	-	-	1M
	Akyurt (1F)	-	-	1F
	Elmadağ (1M)	-	-	1M
	Gölbaşı (1M)	-	-	1M
	Kızılcahamam (1F)	-	-	1F
	Polatlı (1M)	-	-	1M
***Haemaphysalis parva*** ** (35)**	Central (9M, 17F)	2F	2M	5M, 13F
	Akyurt (1M, 1F)	-	-	1M
	Ayaş (1F)	-	-	1F
	Çubuk (2F)	-	-	1F
	Çamlıdere (1F)	-	-	-
	Gölbaşı (1F)	-	-	1F
	Haymana (1F)	-	-	-
	Kızılcahamam (1F)	-	-	-
***Haemaphysalis punctata*** ** (3)**	Central (3M)	1M	-	-
***Haemaphysalis*** ** spp. (nymph) (1)**	Central (1M)	-	-	-
***Rhipicephalus turanicus*** ** (28)**	Central (14M, 7F)	-	-	-
	Bala (1F)	-	-	-
	Gölbaşı (1M)	-	-	-
	Güdül (1M)	-	-	-
	Kazan (2F)	-	-	-
	Kızılcahamam (2M)	-	-	-
***Rhipicephalus bursa*** ** (3)**	Central (2F)	-	-	-
	Çubuk (1F)	-	-	-
***Rhipicephalus sanguineus*** ** (3)**	Central (1M, 2F)	-	-	-
***Ixodes ricinus*** ** (2)**	Çamlıdere (1F)	-	-	-
	Kızılcahamam (1F)	-	-	-
**Total 169**	**84M (49.7%), 78F (46.1%), 7N (4.1%)**	**4 (2.3%)**	**6 (3.5%)**	**46 (27.2%)**

F, female; M, male; N, nymph.

*Unfed ticks: The ticks removed from humans were obtained as engorged nymphs and were then allowed to molt to the adult stage.

A total of 169 ticks, 35 *Ha. parva*, 30 *H. marginatum*, 28 *Rh. turanicus*, 25 *D. marginatus*, 17 *H. excavatum*, 16 *H. aegyptium*, 6 *Hyalomma* spp. (nymphs), 3 *Ha. punctata*, 3 *Rh. bursa*, 3 *Rh. sanguineus*, 2 *I. ricinus*, and 1 *Haemaphysalis* spp. (nymph), were collected from humans in different parts of Ankara (Table 1). Amongst the collected ticks, 8 *H. aegyptium*, 1 *H. excavatum*, and 1 *H. marginatum* were obtained as engorged nymph and were then allowed to molt to the adult stage (as unfed ticks) under suitable conditions. Ticks collected in this study have been partially fed except the unfed 10 ticks. The number of the ticks found per patient is one. 55 ticks were found to be infected with at least one of the pathogens in the genera *Babesia*, *Borrelia*, or *Rickettsia*. Most of the ticks were infected with a single pathogen; however, one tick (*Ha. parva*) contained the DNA of both *Babesia* spp. and *Rickettsia* spp. in a mixed infection. The regarding information is summarized in Table 1.

**Figure 2:**
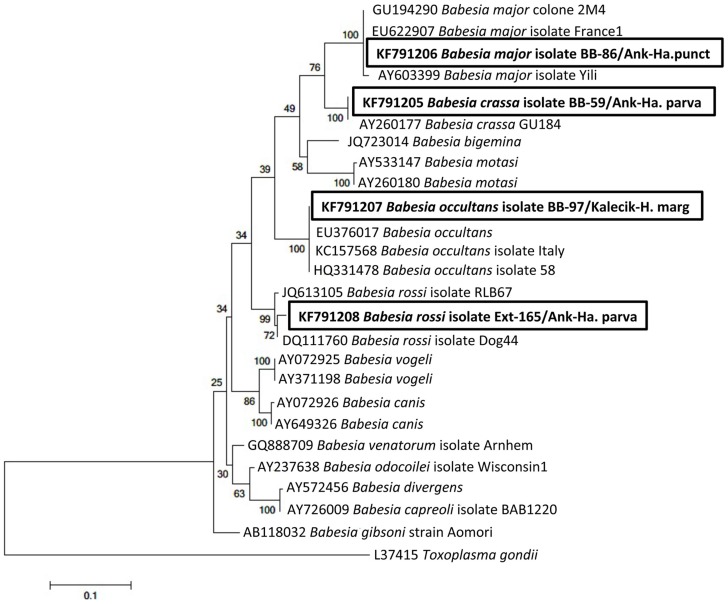
Phylogenetic tree based on aligned sequences of *18S rRNA* gene of *Babesia* spp. with *Toxoplasma gondii* as outgroup and constructed by using Maximum Likelihood method calculated under the GTR+I+G substitution model in MEGA5.1 software. The babesial sequences obtained in this study are shown in a bold font. GenBank accession numbers of sequences and names of lineages are given before species names.

**Figure 3:**
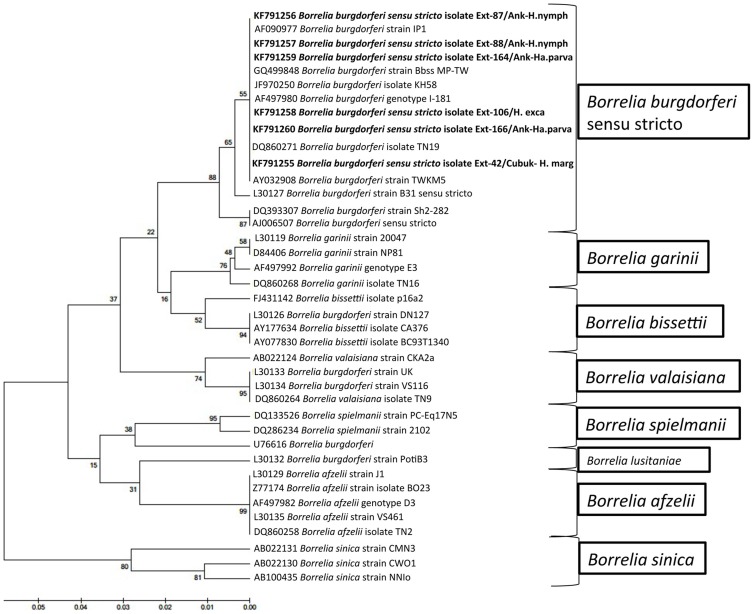
Phylogenetic tree based on aligned sequences of *5S-23S rDNA* intergenic spacer region of *Borrelia burgdorferi* sensu lato and constructed by using the UPGMA method in MEGA5.1 software. The *Borrelia* sequences obtained in this study are shown in a bold font. GenBank accession numbers of sequences and names of lineages are given before species names.

**Figure 4:**
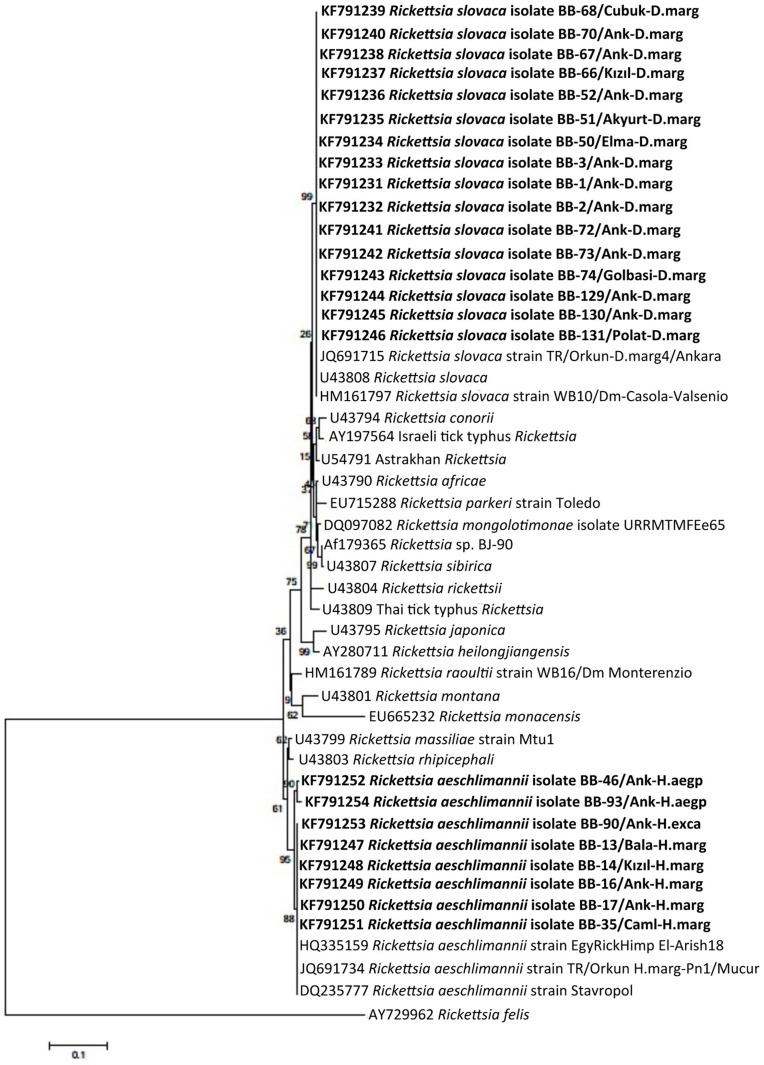
Phylogenetic tree based on aligned sequences of the rickettsial *ompA* gene and constructed by using the Neighbor-Joining method in MEGA5.1 software. The rickettsial sequences obtained in this study are shown in a bold font. GenBank accession numbers of sequences and names of lineages are given before species names.

**Figure 5:**
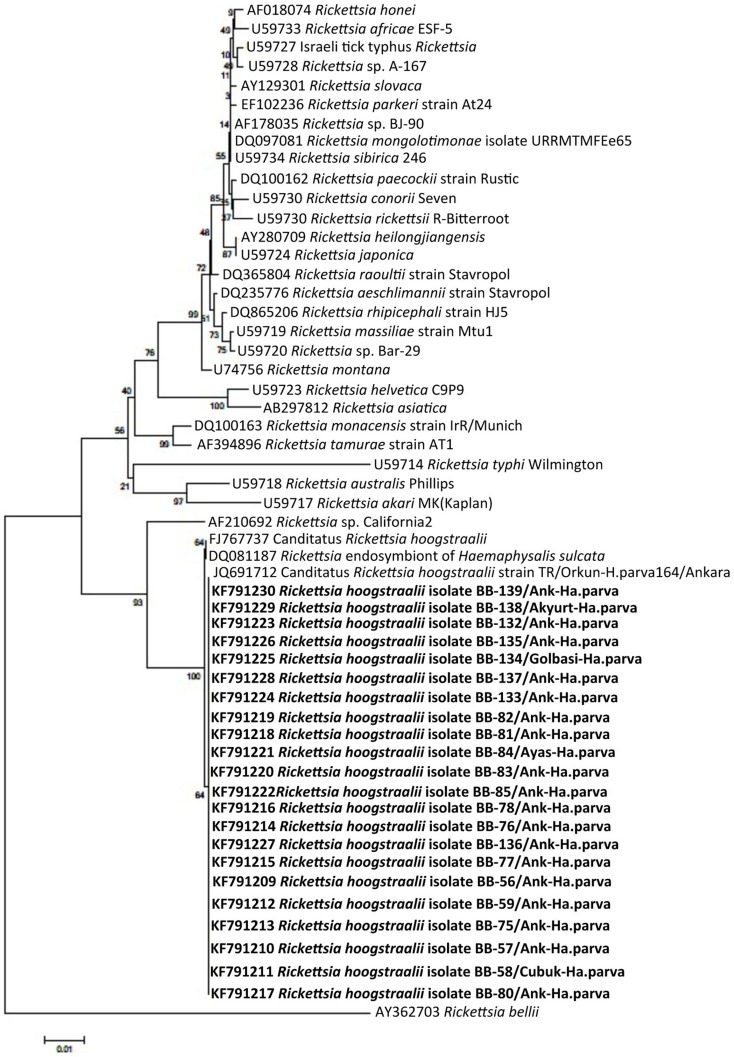
Phylogenetic tree based on aligned sequences of the rickettsial *gltA* gene and constructed by using the Neighbor-Joining method in MEGA5.1 software. The rickettsial sequences obtained in this study are shown in a bold font. GenBank accession numbers of sequences and names of lineages are given before species names.

Every DNA sample was found to be positive for the tick *16S rDNA* and was subjected to PCR assay to detect tick-borne pathogens. Additionally, pre-PCR tests, which are performed to determine the sensitivity of PCR assay, showed that even the lowest copies of bacterial and protozoan genes are yielded visible positive bands. As a result of the PCR analyses, *Babesia* spp. was detected in 4 tick specimens (2.3%). *18S rRNA* nucleotide sequence indicated that *B. crassa*, *B. major* and *B. rossi* were found in *Ha. parva*, *Ha. punctata* and *Ha. parva* tick individuals, respectively, attached to humans in central Ankara. Among these, one *H. parva* infected with *B. crassa* was also infected with *R. hoogstraalii*. Additionally, *B. occultans* was detected in 1 *H. marginatum* to a person attached in Kalecik. *5S-23S rDNA* IGS nucleotide sequences derived from *Borrelia* spp. positive ticks displayed that *B. burgdorferi* sensu stricto was found in 6 tick specimens (3.5%): 1 *H. marginatum* attached in Çubuk, 1 *H. excavatum*, 2 *Hyalomma* spp. (nymph) and 2 *Ha. parva* attached in Central Ankara. Furthermore, rickettsial DNA was detected in 46 ticks (27.2%). *Rickettsia* spp. was determined as the most prevalent tick-borne pathogen in this study. *OmpA* gene sequence analysis showed that *R. aeschlimannii* was detected in 5 *H. marginatum* attached in Bala, Çamlıdere, Kızılcahamam and central Ankara, 1 *H. aegyptium* attached in Kazan and 1 unfed *H. aegyptium*, which was originally obtained as engorged nymph from a person and then allowed to molt to the adult stage as described above in central Ankara and 1 *H. excavatum* attached in central Ankara. 16 *D. marginatus*, which makes 64 percent of the total *D. marginatus* ticks, attached in Akyurt, Elmadağ, Çubuk, Gölbaşı, Kızılcahamam, Polatlı and central Ankara were found to be infected with *R. slovaca*. According to the *gltA* nucleotide sequences (we could not obtain PCR products from the *ompA* gene), *R. hoogstraalii* was detected in 22 of 35 *Ha. parva* ticks attached in Ayaş, Akyurt, Çubuk, Gölbaşı, and central Ankara. We could not detect DNA of *Babesia* spp., *Borrelia* spp., or *Rickettsia* spp. in *Haemaphysalis* spp. nymph, *I. ricinus*, *Rh. bursa*, *Rh sanguineus* or *Rh. turanicus* specimens. Detailed information about the tested ticks and the nucleotide similarities of the obtained *Babesia*, *Borrelia*, and *Rickettsia* sequences in this study are given in Table 1 and 2, respectively. In addition, Phylogenetic trees were constructed separately by using *18S rRNA* gene of *Babesia* spp., *5S-23S rDNA* IGS of *B. burgdorferi* sensu lato and *ompA* and *gltA* genes of *Rickettsia* spp. are illustrated in Figs. 2–5.

**Table 2:** Turkish *Babesia*, *Borrelia*, and *Rickettsia* spp. detected in this study and their level of nucleotide similarity with other strains.

Detected pathogens		Sequenced gene	Tick species (No. positive ticks)	Nucleotide identity percentage	GenBank accession no.
***Babesia*** ** spp.**	*Babesia crassa*	*18S rRNA*	*Haemaphysalis parva*	100^a^	KF791205
	*Babesia major*	*18S rRNA*	*Haemaphysalis punctata*	100^b^	KF791206
	*Babesia occultans*	*18S rRNA*	*Hyalomma marginatum*	100^c^	KF791207
	*Babesia rossi*	*18S rRNA*	*Haemaphysalis parva*	98.9^d^	KF791208
***Borrelia burgdorferi*** ** sensu lato**	*Borrelia burgdorferi* sensu stricto	*5S-23S rDNA*	*Hyalomma marginatum*	100^e^	KF791255
		*5S-23S rDNA*	*Hyalomma excavatum*	100^e^	KF791258
		*5S-23S rDNA*	*Hyalomma* spp. (nymph) (2)	100^e^	KF791257, 58
		*5S-23S rDNA*	*Haemaphysalis parva* (2)	100^f^^, ^ g	KF791259, 60
***Rickettsia*** ** spp.**	*Rickettsia aeschlimannii*	*ompA*	*Hyalomma marginatum* (5)	100^h^	KF791247–51
		*ompA*	*Hyalomma aegyptium* (2)	100ı	KF791252, 54
		*ompA*	*Hyalomma excavatum*	99.8^h^	KF791253
	*Rickettsia slovaca*	*ompA*	*Dermacentor marginatus* (16)	99.8–100^j^^, ^ k	KF791231–46
	*Rickettsia hoogstraalii*	*gltA*	*Haemaphysalis parva* (22)	100^l^	KF791209–30

a *Babesia crassa* GU184 accession no. AY260177.

b *Babesia major* isolate France1 accession no. EU622907.

c *Babesia occultans* isolate Italy-366/12-20 accession no. KC157568 and *Babesia occultans* isolate 58 accession no. HQ331478.

d *Babesia canis rossi* isolate Dog-44 accession no. DQ111760.

e *Borrelia burgdorferi* isolate TN19 accession no. DQ860271.

f *Borrelia burgdorferi* N40 accession no. CP002228.

g *Borrelia burgdorferi* genotype I-181 accession no. AF497980.

h *Rickettsia aeschlimannii* strain EgyRickHimp-El-Arish-17 accession no. HQ335158.

ı *Rickettsia aeschlimannii* strain TR/Orkun-H.aegyp86/Ankara accession no. JQ691728.

j *Rickettsia slovaca* 13-B accession no. CP002428.

k *Rickettsia slovaca* strain TR/Orkun-D.marg79/Ankara accession no. JQ691724.

l Candidatus *Rickettsia hoogstraalii* strain TR/Orkun-H.parva164/Ankara accession no. JQ691712.

## Discussion

Ticks transmit a great variety of viral, bacterial (including rickettsial) and protozoan disease agents so that they play a major role in the epidemiology of tick-borne diseases affecting both human and animals. The incidence and recognition of ticks and tick-borne diseases increases steadily year by year worldwide [1], [2], [7]. The increasing outbreaks in recent years are the evidence of this situation [4]. In Turkey, TBDs such as Lyme-borreliosis and spotted fever group rickettsiosis for humans and babesiosis for both animals and humans have a remarkable importance [8]–[10], [12], [30]–[35].

In the present study, we have retrieved new data related to both some tick-borne pathogens and its vectors. The most striking of these is that we have detected *B. rossi* in *Ha. parva* in Turkey. *Babesia rossi*, the most pathogenic *Babesia* species of dogs, is transmitted by *Haemaphysalis* ticks (mainly *Ha. leachi*) and prevalent in South Africa and other African countries, such as Nigeria and Sudan [36]. Interestingly, *B. rossi* was detected in one *Ha. parva* removed from humans in central Ankara in this study. According to *18S rRNA* sequence, our Turkish *B. rossi* sequence has a 98.9% similarity with the *B. rossi* isolate Dog-44 (accession no. DQ111760) obtained from a dog in Sudan (Table 2). This is the first report of the presence of the native-*B. rossi* in a country that is outside of the African continent and in a *Ha. parva* tick. Furthermore, we detected *B. crassa*, a large *Babesia* spp. of small ruminants, in a *Ha. parva* tick. *Babesia crassa* was first detected from a sheep in Iran [37], but no detailed information about the distribution and vectors of this species was given. According to the sequence analysis, the *B. crassa* sequence detected in this study was 100% similar to the *B. crassa* (accession no. AY260177) obtained from a sheep in Turkey (Table 2). Although the other *B. crassa* was detected from a sheep previously in Turkey, the detected area information was not provided [38]. However, it is clear that *B. crassa* exists in both ticks and sheep in Turkey. Our new findings also point out the existence of *B. crassa* in *Ha. parva*. Interestingly, the *B. crassa* sequence detected in this study has 95% similarity with *B. crassa* (accession no. AY260176) that was detected from the Turkish border of Iran. Schnittger et al. (2004) stressed that it is necessary to design strain-specific oligonucleotides allowing the discrimination between *B. crassa* (Iran) and *B. crassa* (Turkey) [39]. More detailed studies towards *B. crassa* and a large number of sequences are required to determine the difference between both *Babesia* and to come to the conclusion whether we come across a variant of *B. crassa*. *Babesia occultans*, which is moderately pathogenic bovine *Babesia* species, was detected in *H. marginatum*. According to the sequence analysis, the Turkish *B. occultans* sequence was 100% similar to the *B. occultans* isolate Italy-366/12-20 (accession no. KC157568) detected from the cattle in Italy and *B. occultans* isolate 58 (accession no. HQ331478) detected from *H. marginatum* in Tunisia (Table 2). *Babesia occultans*, which is transmitted by mainly *Hyalomma* spp., is prevalent in Africa. It is thought to be a non-pathogen or of low pathogenicity [36], [40]. On other hand, a clinical outbreak caused by *B. occultans* has recently been reported in cows that displayed fever, anemia, and severe alteration in the hematological parameters in Italy [41]. Mean while, there are no clear reports about the existence of *B. occultans* in Turkey, although the similar sequences, *Babesia* sp. Kayseri 1 (accession no. EF434786) [42] and *Babesia* sp. H4 (accession no. JF923655) [9] obtained from *H. marginatum* ticks collected from cattle and *Babesia* sp. CS58 (accession no. EU622824) obtained from cattle [32], were reported in previous studies. These sequences are not enough for a definitive identification; therefore, we could not compare our *B. occultans* sequence with the others. However, our study clearly shows the presence of *B. occultans* in Turkey. Additionally, we detected *B. major*, a low-pathogenic bovine babesia species, in *Ha. puctata*. According to the sequence analysis, the Turkish *B. major* has 100% similarity with the *B. major* isolate France 1 (accession no. EU622907) which was obtained from cattle in France (Table 2). The presence of *B. major* was reported from the cattle previously in East Black Sea Region of Turkey [32]. However, this is the first report of the presence of *B. major* in a tick. The nucleotide similarities of sequences and phylogenetic relationships are shown in detail in Table 2 and Fig. 2, respectively.

*Borrelia burgdorferi* sensu stricto, which is the primary pathogenic genospecies of Lyme disease [43], was detected in one *H. marginatum*, 2 *Hyalomma* spp. (nymph), 2 *Ha. parva* and one *H. excavatum*. According to *5S-23S rDNA* IGS sequence analysis, *B. burgdorferi* sensu stricto sequences obtained in this study are 100% similar to the reference strains (*B. burgdorferi* N40, accession no. CP002228, isolate TN19, accession no. DQ860271 and genotype I-181, accession no. AF497980). More detailed data and a phylogenetic tree are given in Table 2 and Fig. 3, respectively. Lyme borreliosis, transmitted mainly by ticks belonging to *Ixodes* genus, is the most common tick-borne zoonosis in the temperate zone of the northern hemisphere [44]. In Turkey, *B. burgdorferi* sensu stricto was hitherto isolated from only one unfed *I. ricinus* collected from Istanbul in 2003 [31]. However, non-*Ixodes* spp. ticks were to be infected with *B. burgdorferi* sensu stricto in this study. This indicated that either the ticks ingested infected body fluids or may be capable to transmit this bacterium (potential vectors). Yet, we cannot clearly say that these tick species are the vector for *B. burgdorferi* sensu stricto with the current data. It is possible that the humans had bacteremia of *B. burgdorferi* sensu stricto and this pathogen passed to the ticks during the feeding process or the ticks might have been infected in previous life stages, as well. As noted by Kahl et al. (2002): “to be considered a vector, a tick species must: (1) feed on infectious vertebrates; (2) be able to acquire the pathogen during the blood meal; (3) maintain it through one ore more life stages (transstadial passage); and (4) pass it on to other host when feeding again. Otherwise it is a non-vector tick” [45]. Therefore, more detailed experimental studies are required to determine the vector competence of these tick species. Nevertheless, it is already evident that *B. burgdorferi* sensu stricto is circulating in Ankara, because we did not record any history of a tick bite outside of Ankara from the patients. Hence, Lyme disease should be taken into consideration in patients who had a tick bite in Ankara.

Spotted fever group rickettsiae have recently been found with remarkable infection rates in ticks in Turkey [10], [12]. Likewise, spotted fever group rickettsiae were the most commonly observed agents with 27.2% in this study (Table 1). Two human pathogenic rickettsial species (*R. aeschlimannii* and *R. slovaca*) and one species (*R. hoogstraalii*) with unknown pathogenicity were detected in ticks. Among them, *R. aeschlimannii* is transmitted mainly by *Hyalomma* ticks [46]. This bacterium was found in 5 *H. marginatum*, 2 *H. aegyptium*, and 1 *H. excavatum* in this study. As a result of the *ompA* gene sequence, *R. aeschlimannii* sequences obtained in this study are by 99.8–100% similar to the reference strains (*R. aeschlimannii* strain EgyRickHimp-El-Arish-17, accession no. HQ335158 and strain TR/Orkun-H.agyp86/Ankara accession no. JQ691728). In Turkey, *R. aeschlimannii* was found in 5 *H. aegyptium*, 2 *H. marginatum*, and one *Rh. bursa* collected from humans in Istanbul [10]. In our previous study, this pathogen was detected (in 32% of *Rickettsia*-positive ticks) in 3 *H. marginatum*, 2 *H. aegyptium* (unfed), 2 *Hyalomma* spp. (nymph), 1 *Rh. turanicus* collected from humans and animals in Kırşehir and Ankara [12]. These results show that our findings are in parallel with the previous findings. *Rickettsia slovaca*, the etiological agent of Tick-borne lymphadenopathy/*Dermacentor*-borne necrosis erythema and lymphadenopathy (TIBOLA/DEBONEL) [47], was detected in 16 *D. marginatus*, which are 64 percent of the total *D. marginatus* ticks (Table 1). According to *ompA* sequence, *R. slovaca* sequences detected in this study have 99.8–100% similarity with the reference strains (*R. slovaca* 13-B, accession no. CP002428, and strain TR/Orkun-D.marg79/Ankara accession no. JQ691724). Previously, we had detected this bacterium in 8 *D. marginatus* (80% of total *D. marginatus* ticks) collected from human and cattle in Ankara [12]. As a result, we again stress that *R. aeschlimannii* and *R. slovaca* are significant disease agents for humans and should not be neglected in this area. The clinical signs caused by these pathogens should be considered in the differential diagnosis of the tick-borne diseases in patients. Additionally, we detected *R. hoogstraalii*, a rickettsia with an unknown pathogenicity [48], in 22 out of 35 *Ha. parva* ticks collected (Table 1). According to the *gltA* gene sequence, the sequences obtained from this study are 100% similar to *R. hoogstraalii* strain TR/Orkun-Ha.parva164/Ankara (accession no JQ691712). In Turkey, *R. hoogstraalii* had been detected in 4 *Ha. parva* collected from humans in Ankara in our previous study [12]. The pathogenicity of *R. hoogstraalii* is unknown, but this rickettsia is widespread in *Ha. parva* ticks in this area. The nucleotide similarities of rickettsial sequences detected in this study and phylogenetic relationships are shown in detail in Table 2 and Figs. 4–5, respectively.

In conclusion, we have studied a broad range of tick-borne pathogens and detected several pathogens in ticks removed from humans in Ankara. Moreover, this study shows the presence of new tick-borne pathogens in Turkey. Among them, *B. rossi*, especially, was only known to be prevalent in Africa. However, this pathogen was detected in a Turkish tick and this finding reported here is the first evidence of the existence of *B. rossi* in a country outside of Africa. This disease agent, which is very pathogenic for dogs, must be considered in this area, because our results tell us that it is likely that *B. rossi* has a wide distribution in Turkey. Hence, more detailed epidemiologic studies are required in the future. Additionally, we detected *B. crassa*, of which we have very limited information on its distribution and vector, in a *Ha. parva* tick. Moreover, there is no clear information about the presence of *B. occultans* in Turkey, which we detected again in our study. *Babesia major* was also detected in ticks. Although we could not detect human pathogenic *Babesia* species, very significant epidemiological data related to animal babesiosis were collected in this study. Furthermore, we detected two human pathogenic rickettsia species (*R. aeschlimannii* and *R. slovaca*) which were previously detected in the same area with a high prevalence. We recommend that these pathogens should be taken into consideration in patients who had a tick bite in Turkey, especially in Ankara. Additionally, *B. bugdorferi* sensu stricto was detected in different tick species (non-*Ixodes* spp.). This pathogen is transmitted by mainly *I. ricinus*, but this tick is not common among tick species in Ankara due to the existence of very restricted suitable habitat for *I. ricinus* ticks [16]. This situation also opens up the discussion of whether the other vector or carrier tick species exist in the region. However, we do not know the vector competence of these tick species at the moment without detailed experimental studies. It is important to point out that this pathogen caused Lyme disease exists in the region and should not be overlooked in patients exposed to tick bites.
